# Update of the japanese national registry of pediatric rheumatic disease

**DOI:** 10.1186/1546-0096-12-S1-P294

**Published:** 2014-09-17

**Authors:** Ken-Ichi Yamagchi, Yasuhito Nerome, Tomoyuki Imagawa

**Affiliations:** 1 Immuno-Rheumatology Center, St. Luke's International Hospital, Tokyo, Japan; 2 Pediatrics, Kagoshima University, Kagoshima, Japan; 3 Dpartment of Immunology and Infectious Diseases, Kanagawa Children's Medical Center, Yokohama, Japan

## Introduction

We, Pediatric Rheumatology Association of Japan (PRAJ), have set up new online national registry of pediatric rheumatic diseases.

## Objectives

We report the first several months of enrollment to PRICURE (Pediatric Rheumatology International Collaboration Unit Registry).

## Methods

Professor Shuji TAKEI, the former chairman of PRAJ had ordered his working group to set up a new registry system in 2012. Ethical issues about the registry in which we use clinical information were discussed by ethics committee of PRAJ and it had been approved in 2013. We started to collected basic information (e.g. name of rheumatic disease, age of onset, sex, clinical course) and further information (e.g. clinical symptoms, laboratory examination abnormalities, radiological examination abnormalities, treatment, complications) by web-based way in spring 2014.

## Results

In the end of May 2014, 394 cases had been registered in the PRICURE. The number of cases of each diseases are as follow, Juvenile Idiopathic Arthritis 174, Systemic Lupus Erythematosus 65, Juvenile Dermatomyositis 43, Sjogren syndrome 14, Mixed Connective Tissue Disease 29, Systemic Sclerosis 12, Behcet’s Disease 8, Vasculitis 16, Autoinflammatory Syndromes 1, Fibromyalgia 38.

## Conclusion

We hope that this registry contribute to the development of clinical research in the field of pediatric rheumatology.

## Disclosure of interest

None declared

